# High dose vitamin D supplementation decreases the risk of deficiency in male conscripts, but has no effect on physical performance—A randomized study

**DOI:** 10.1002/jeo2.12023

**Published:** 2024-05-01

**Authors:** Leho Rips, Alar Toom, Rein Kuik, Ahti Varblane, Hanno Mölder, Ragnar Kibur, Marika Laidvere, Mart Kull, Jüri‐Toomas Kartus, Helena Gapeyeva, Madis Rahu

**Affiliations:** ^1^ Sports Medicine and Rehabilitation Clinic Tartu University Hospital Tartu Estonia; ^2^ Department of Sports Medicine and Rehabilitation, Institute of Clinical Medicine, Faculty of Medicine University of Tartu Tartu Estonia; ^3^ Centre of Military Disaster Medicine Estonian National Defence College Tartu Estonia; ^4^ Department of Orthopedics Central Finland Central Hospital Keskussairaalantie 19 Jyväskylä 40620 Finland; ^5^ Medical Centre of the 2nd Infantry Brigade CSS Battalion Estonian Defence Forces Võru Estonia; ^6^ Department of Nursing and Midwifery Tartu Health Care College Tartu Estonia; ^7^ Viljandi Hospital Viljandi County Estonia; ^8^ Institute of Clinical Sciences, Sahlgrenska Academy University of Gothenburg Göteborg Sweden; ^9^ Clinic of Medical Rehabilitation East‐Tallinn Central Hospital Tallinn Estonia

**Keywords:** APFT physical test, hand grip, military training, supplementation, vitamin D

## Abstract

**Purpose:**

Physical load during military training might increase the need for vitamin D; therefore, supplementation could be beneficial for 25(OH)D serum levels and physical performance.

**Methods:**

One hundred and twelve male conscripts were randomized into two vitamin D oil capsule supplementation groups: 55 participants in the 600 IU group and 57 in the 4000 IU group with a follow‐up period from July 2021 to May 2022. Physical fitness tests were performed in July, October and May. Hand grip strength tests were performed in July, October and January. Blood serum (25(OH)D), parathyroid hormone PTH), calcium and ionized calcium (i‐Ca) values were measured in July, October, January and May.

**Results:**

The 600 IU group had a lower (*p* < 0.001) value of 25(OH)D at all time points compared to the 4000 IU group, except at baseline. None of the subjects in the 600 IU group reached sufficient levels of 75 nmol/L of 25(OH)D in January and May. In May, 60% of participants in the 600 IU group and 30% in the 4000 IU group had 25(OH)D levels under 50 nmol/L. No significant differences in PTH or i‐Ca values were found between the study groups at any time point. No significant differences at any time point were found in the physical fitness test or hand grip strength test between the groups.

**Conclusion:**

A 10‐month vitamin D supplementation with 4000 IU decreased the incidence of vitamin D deficiency (<75 nmol/L) in young, male army conscripts during wintertime, but no differences in physical performance were found compared to 600 IU supplementation.

**Level of Evidence:**

Level I, Prospective randomized study.

**Abbreviations**
25(OH)D: 25‐hydroxyvitamin D
APFT: Army Physical Fitness Test
BMI: body mass index
Ca: calcium
EFSA: European Food Safety Authority
ES: Endocrine Society
i‐Ca: ionized calcium
n.s.: non‐significant
PTH: parathyroid hormone
RCT: randomized controlled study
RDA: Recommended Dietary Allowance
SD: standard deviation

## INTRODUCTION

There is a growing interest in vitamin D deficiency and its consequences to human health among the general population, as well as among individuals participating in high‐demand activities such as military service [12, 13, 40]. Recent studies have shown that vitamin D deficiency is common in the Nordic countries (above latitude 59°N) [22, 35], but also in regions with high sun exposure [24]. In military service, many specific conditions, such as geographic region, uniform/camouflage clothing, training conditions, weather and environmental conditions (e.g., submarine service and type of shelter) could put soldiers at higher risk of vitamin D deficiency [40]. Several studies have shown a high prevalence of vitamin D deficiency among military personnel in different regions of the world [10, 17, 28, 32].

Natural sunlight with ultraviolet UVB irradiation (wavelength 290–320 nm) is the main source of endogenous vitamin D synthesis via naked skin [14]. Skin pigmentation, clothing covering the skin, regular use of topical sunscreens, ageing and the lower zenith angle of the sun can negatively affect the skin's ability to produce vitamin D [18]. Intake of cod liver oil, fish, eggs and meat are well‐known exogenous sources of vitamin D3 and mushrooms for Vitamin D2 [18]. Vitamin D fortification of milk and cheese is common practice in the Nordic countries [23]. Supplements in the form of capsules, tablets, sprays and different multivitamin mixtures are widely available on the market today.

Measurement of the form 25‐hydroxyvitamin D (25(OH)D) in blood serum is most often used to assess levels of vitamin D in humans [5]. The normative values of vitamin D are still undetermined. Serum 25(OH)D values for deficiency are defined as <20 or <50 nmol/L and sufficiency >30 ng/mL or >75 nmol/L, according to the Endocrine Society [18]. Nordic Nutrition Recommendations gives 50 nmol/L as a sufficient value of 25(OH)D in adults [23]. Controversially, Bischoff‐Ferrari et al. [3] state that the serum concentration of 25(OH)D should not be below 75 nmol/L, and the target is to have values between 90 and 100 nmol/l. Also, an optimal blood serum 25(OH)D level is important for the suppression of parathyroid hormone (PTH) [3]. In the studies by Rips et al., levels <25 nmol/L were determined as severe deficiency, <50 nmol/L as deficiency, <75 nmol/L as insufficiency and >75 nmol/L as a sufficient level [35]. The negative consequences of vitamin D deficiency have been well‐known for decades. Ricket's disease, osteomalacia, osteoporosis, stress fractures, higher risk of traumatic fractures in the elderly population and a higher incidence of hypertension and cardiovascular diseases are all associated with vitamin D deficiency. Furthermore, depression and decreased physical fitness have also been found to be related to vitamin D deficiency [9, 18, 23].

Studies have found that there is a high risk of vitamin D deficiency in the Nordic countries among the general population. In an Estonian‐population‐based study, Kull et al. [21] found mean winter serum 25(OH)D levels of 43.7 nmol/L. In a randomized controlled study (RCT) by Rips et al. [36], the lowest mean 25(OH)D value of 21.9 nmol/L was found in March in a placebo group of young men undergoing military service. Another Estonian study found critically low vitamin D values (<25 nmol/L) in 36% and deficiency (<50 nmol/L) in 92% of young conscripts in the spring season [35].

Daily intake of vitamin D and supplementation is currently under discussion and there is lack of consensus. The European Food Safety Authority (EFSA) panel's suggestion for adults is an intake of 15 µg (600 IU) in order to reach a range between 34 and 91 nmol/L [9]. The same daily intake of 600 IU is suggested by the Nordic Nutrition Recommendations panel [23]. Rips et al. [36] found that daily winter‐ and springtime supplementation with 1200 IU for seven months in young male conscripts resulted in a mean maximum 25(OH)D value of 59.8 nmol/L, which was slightly reduced during springtime and had a lowest mean value of 50.2 nmol/L in March. The Endocrine Society Committee suggests a daily supplementation between 1500 and 2000 IU for those in the population at risk of deficiency, and it suggests an upper limit of 4000 IU in young men [18].

Among professional sportsmen, women and military personnel, there is growing interest in the role of vitamin D on physical fitness and general health [6, 29]. There is evidence that vitamin D deficiency is a risk factor for stress fractures and defects in the bone healing process [34], and long‐lasting vitamin D deficiency could lead to osteopenia and osteoporosis [18], and it has been shown that vitamin D supplementation can have a positive influence on bone health [40]. However, there is still lack of proof for the positive effect of vitamin D supplementation on physical fitness in those people with high physical demands [29]. In the study of Heileson et al. [15], a correlation between physical fitness and vitamin D deficiency was found. High‐level physical fitness and general good health play a crucial role in potentially life‐threatening situations encountered in military service. There is also evidence suggesting that the seasonal variation in vitamin D levels can affect muscle function [25]. Thus, it appears important to avoid vitamin D deficiency and its related consequences during military service.

For the objective measurement of physical fitness during service in the army, the Army Physical Fitness Test (APFT) is widely used [20]. The APFT was designed in the United States (US) to measure the physical fitness of military personnel and has been used during the last decades in the Estonian Army. The test consists of three different exercises that test the endurance and muscular fitness of the soldier. The hand grip strength test is also widely used to measure upper limb muscle strength; it can also be used as a predictor of general health, and it has been shown that lower strength is associated with vitamin D deficiency in both sexes regardless of age [19, 27].

Based on current evidence, supplementation with 1200 IU vitamin D is not enough to reach sufficient 25(OH)D levels in Estonian conscripts [36]. A prospective, randomized study, with two different supplementation dosages was designed, involving young male conscripts in the Estonian Army to examine the effect of vitamin D supplementation on muscular fitness and 25(OH)D blood serum levels.

The hypothesis of the study was that oral vitamin D supplementation with 4000 IU decreases the risk of vitamin D deficiency risk compared to 600 IU during the winter season and increases physical fitness.

The primary outcome of the study was serum levels of 25(OH)D during the study period. The secondary outcome of the study was physical fitness in terms of physical fitness test results and hand grip strength test.

## METHODS

### Study design and data collection

A longitudinal, triple‐blinded (participants and researchers), randomized, controlled trial (ClinicalTrials.gov NCT04939636) with a 10‐month follow‐up period from July 2021 until May 2022 was performed. The study was approved by the Research Ethics Committee of the University of Tartu no. 323/T‐3, 337M‐25 and 341M‐12 and funded by grant no. KVA‐0.7‐1.1/20/29041 of the Estonian Defense Forces.

### Materials

All conscripts (*n* = 438) entering military service in July 2021 at the Kuperjanov Battalion, Võru, Estonia (situated at a latitude of 58°N, which corresponds to that of southern Alaska in the United States) were asked to participate in the first briefing of this study. The recruits were informed of the purpose of the randomized study, recruitment criteria, and follow‐up methods. Only those who volunteered to participate in the study and provided signed informed consent after this first briefing were included. A total of 135 conscripts initially volunteered to participate, and 116 of them returned their informed consent.

**Figure 1:**
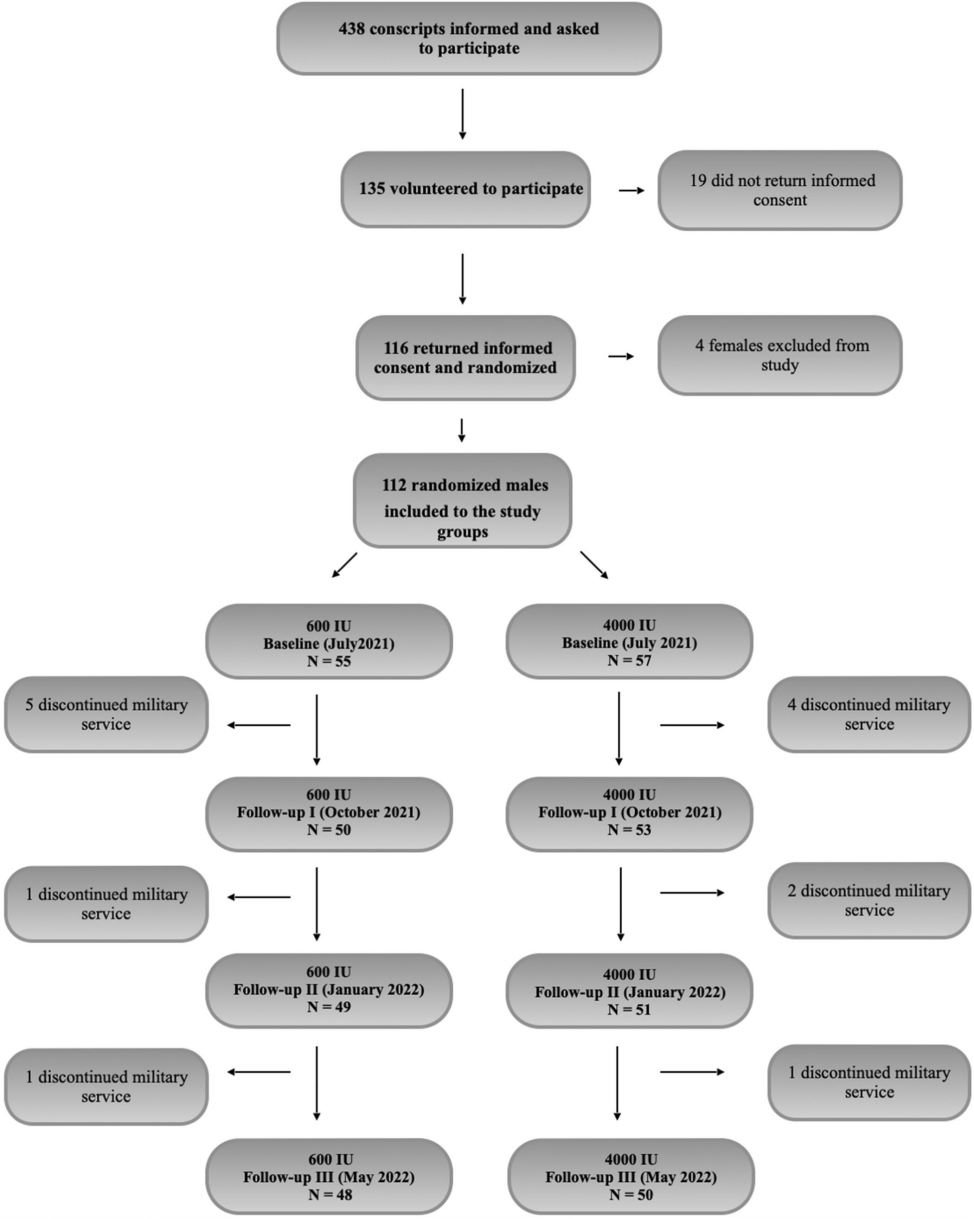
Follow chart of the study.

Four females were excluded from the study, because of the impossibility to standardize physical test results with male participants. Fourteen conscripts—seven in the 600 IU group and seven in the 4000 IU group—were later excluded from the study due to premature cessation of their military service: five for mental health problems, three for lower back pain and six for other medical reasons. Data for 112 male conscripts, all of Caucasian origin, were included in the analysis: 55 in the 600 IU group and 57 in the 4000 IU group. Home‐based Vitamin D supplementation was registered in five conscripts who all were told to discontinue the use at baseline. The anthropometric characteristics of the study groups are presented in Table 1. The exclusion criterion was the inability to continue military service, for any reason. Conscripts who missed test occasions for any reason were registered during the 10‐month follow‐up period. A flow chart of the study is presented in Figure 1.

**Table 1:** Baseline anthropometric data and vitamin D intake.

Characteristics	Anthropometric data			
	Total	600 IU	4000 IU	*p* Value
Number of participants	112	55	57	
Age (years)—Mean (SD)	19.4 (1.13)	19.5 (1.32)	19.3 (0.90)	0.22
Baseline height (cm)—Mean (SD)	182 (5.98)	184 (6.09)	181 (5.74)	0.06
Baseline body mass (kg)—Mean (SD)	79.9 (14.0)	81.6 (14.40)	78.3 (13.60)	0.21
Baseline BMI (kg/m^2^)—Mean (SD)	24.0 (3.90)	24.2 (4.23)	23.7 (3.57)	0.48
Home‐based vitamin D intake at the baseline (yes/no), *n* (%)	5/107 (4)	3/52 (5)	2/55 (4)	‐

Abbreviations: BMI, body mass index; IU, international unit; SD, standard deviation.

In both study groups, the conscripts followed routine military preparations based on the guidelines of the Estonian Defense Forces. Ordinary daily routines start at 6 AM and end at 10 PM with night rest. There are three main meals served and two additional snacks are provided between lunch and dinner. During exercises outside of the military base, tactical food packs are used. Military training in the Estonian Defence Forces starts with 10 weeks of basic training, consisting of physical preparations and special training for different military needs. Physical fitness training is focused on increasing aerobic and anaerobic capacity, varying from a minimum of 0.5–2 h of daily training to a 50 km extreme military‐specific hike in a complex landscape, with full military equipment carried by the conscript. The basic training programme consists of a daily morning run up to 5 km, bodyweight strength training, and maximum muscle strength training in the gym guided by physical preparation coaches. The following eight months consist of special skills training, physical performance training, self‐defence training, urban and forest combat training, and special tactical and weapons training.

### Anthropometric data collection

Body height (cm) of the conscripts was measured once at the baseline and body mass (kg) was measured four times during the study period, at baseline in July 2021, October 2021, January 2022 and May 2022 in the same morning when blood samples were obtained and by the same nurse at the Kuperjanov Battalion medical centre using standardized equipment. Conscript body mass index (BMI) at baseline was calculated as kg/m^2^.

### Randomization

Computed randomization was used to divide conscripts into two groups. Conscripts in both groups received vitamin D3 capsules, but in one group, the daily dosage was 600 IU/15 µg, and in the second group, the daily dosage was 4000 IU/100 µg. Both types of capsules were standardized for size and colour, administered once per day, in the morning before breakfast, for 10 months. Every conscript was provided with a personal copy of the information on the study with explanations on the supplementation. The general and daily supplementation protocols were supervised by a member of local medical staff who was part of the research team and had undergone special training.

### Vitamin D supplementation

Standardized coded packages (three per conscript) and capsules (100 per package) were manufactured on special order by HC CLOVER PS, SL (Spain). No commercial sponsoring was involved. The key to the package code numbers was stored in a computer database until the unblinding of the participants. At the end of the study, all the packages and capsules were collected and destroyed.

Personal protocols were prepared for registration of possible side effects of the Vitamin D supplementation. All participants were informed of the side effect protocols which were kept at the medical centre of Kuperjanov Batallion.

### Army Physical Fitness Test

The APFT is designed to test the endurance, muscular strength, and cardiovascular respiratory fitness of soldiers. In this test, results are scored for three events, 2 min of push‐ups, 2 min of sit‐ups and a 3.2 km (2 mile) run. The results of each event can range from 0 to 100 points. A minimum of 60 points is needed in each event to pass the test; a minimum overall total of 180 points is therefore needed, so total scores to pass the test can range from 180 to 300 points. Scoring is based on sex and age, and the score tables can be found in the Army FM 7‐22 and Department of the Army Form 705, APFT Scorecard [8]. The participants were informed about the rules of the test including minimum and maximum scores with regards to the APFT guidelines. The APFT physical performance testing took place on the same day, under the same conditions on the same running track, and the results were recorded by the same experienced staff of the Kuperjanov Infantry Battalion. Altogether the APFT test was performed three times during the study period, at the baseline in July 2021, in October 2021 and at the end of study in May 2022.

### The hand grip strength test

The hand grip strength test was performed using a validated hydraulic hand dynamometer (Lafayette Instrument Co.). Measurements were taken from each participant in the standing position, arms at the side, not touching the body, with the elbow slightly bent. Each participant squeezed the dynamometer with as much force as possible. The best result of three trials, with pause of about 10–20 s between the trials, was recorded in kg. The same procedure was performed for both hands. The hand grip strength test was performed at the baseline in July 2021, October 2021 and January 2022.

### Blood serum tests

Blood serum values of (25(OH)D) (sufficient >75 nmol/L, insufficient <75 nmol/L, deficiency <50 nmol/L and severe deficiency <25 nmol/L), parathyroid hormone (PTH, normal 1.48–7.83 pmol/L), calcium (Ca, normal 2.15–2.6 mmol/L) and ionized calcium (Ca‐i, normal 1.12–1.32 mmol/L), were measured four times during the study period: first in July 2021 to provide baseline values, then subsequently in October 2021, January 2022 and May 2022. All blood samples were overnight fasting tests, collected on the same day of the week, and all within the same hour, under standardized conditions in the same medical centre. All samples were collected by Synlab, Estonia from the army base in the same morning and transported to the main laboratory using blood sample transport containers.

### Laboratory measurements

Serum samples for clinical chemistry analysis were collected in serum clot activator tubes (BD Vacutainer SST II Advance Plus Blood Collection Tubes, Becton Dickinson and Company). Calcium measurements were performed using the spectrophotometry method (ADVIA® 1800 Clinical Chemistry System, Siemens Healthcare GmbH). Ionized calcium measurements were performed using ion‐selective electrodes (AVL 9180 Electrolyte Analyzer, Roche Diagnostics). The direct chemiluminescent immunoassay method was used for the measurement of PTH (ADVIA Centaur XP, Siemens Healthcare GmbH). Measurements of 25(OH)D were performed using the direct chemiluminescent immunoassay method (LIAISON XL, DiaSorin S.p.A). All analyses were performed by Synlab.

### Statistical analysis and power calculation

The blood serum values, physical test results, and hand grip strength in the study groups were described by means and standard deviations (SDs). Differences in mean values of the variables were evaluated using a two‐way mixed measures ANOVA test with study group and time being the independent variables. Post hoc testing was done using a Tukey's test with estimated marginal means and Bonferroni correction within and between groups. The correlation between body weight and serum 25(OH)D levels was tested using a correlation test with Pearson correlation coefficients. The distribution of categorical variables was described using absolute numbers and percentages. Statistical significance was set at *p* < 0.05. The primary variable of the study was the level of 25(OH)D in the serum. Based on the findings in the study of Rips et al. [36], a difference of 20 nmol/L between the study groups was considered meaningful to measure. For example, for an SD of 25 nmol/L, 26 participants would be needed in each group to reach a power of 80%. Initially, 112 participants were included in the study to increase the power and to allow for dropouts. For the assessment of outliers and the normality of data distribution, box plots and Shapiro–Wilks tests were used. The homogeneity of variances was checked using Levene's test. In instances where the assumptions of parametric mixed ANOVA were not met, a robust alternative using trimmed means (bwtrim method) was applied.

## RESULTS

The highest value of serum 25(OH)D at baseline was 187 nmol/L in the 600 IU group and 116 nmol/L in the 4000 IU group. The lowest value of serum 25(OH)D at baseline was 28.4 nmol/L in the 600 IU group and 35.7 nmol/L in the 4000 IU group. The 600 IU group had significantly lower mean values of serum 25(OH)D at all time points during the study (*p* < 0.001), except at baseline.

**Figure 2:**
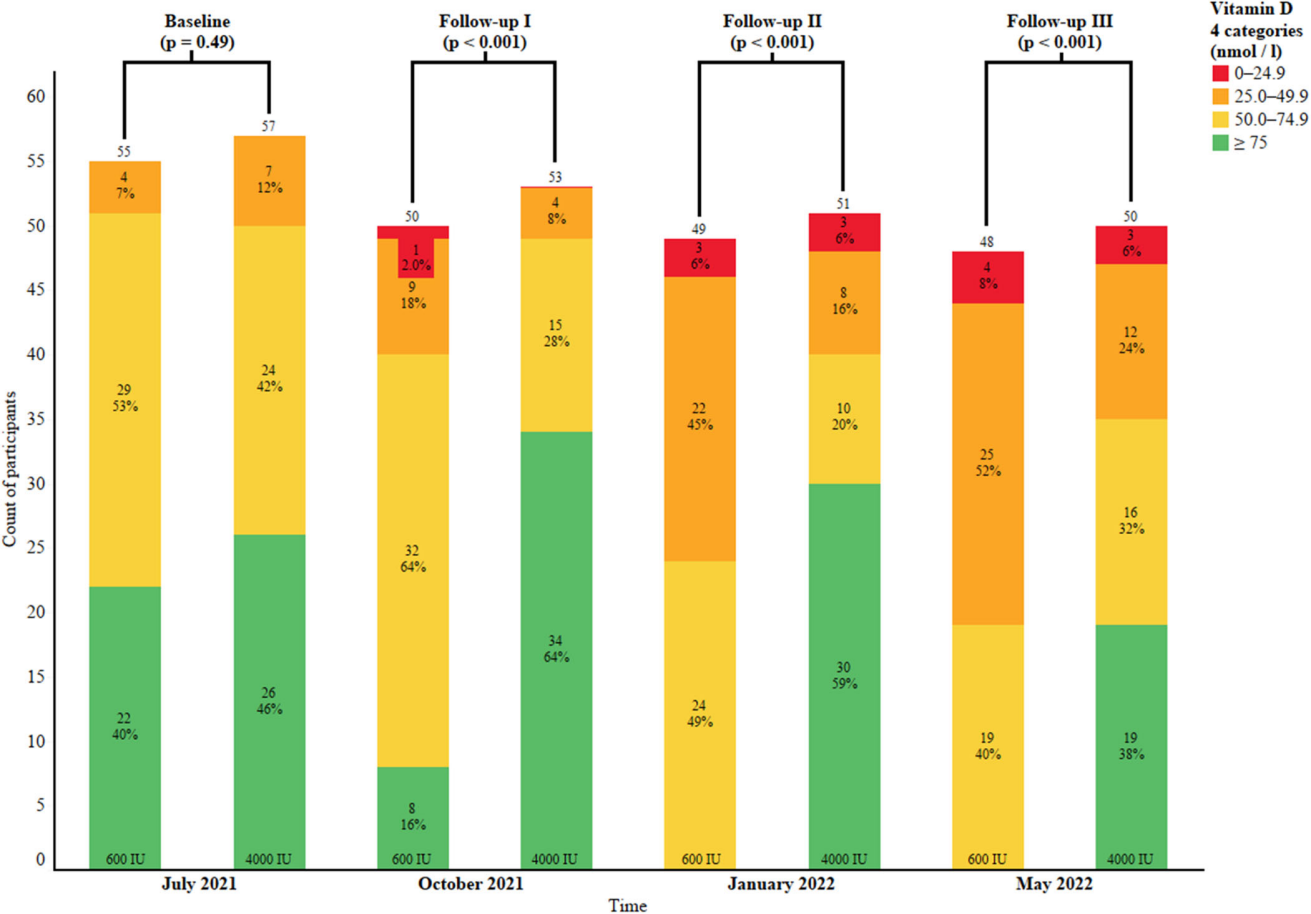
Distributions of serum 25(OH)D levels in both study groups.

The seasonal within‐group variations in serum 25(OH)D results are presented in Table 2. The full seasonal distributions of serum 25(OH)D levels in both study groups are presented in Figure 2. None of the study subjects in the 600 IU group reached a sufficient level (75 nmol/L) of serum 25(OH)D in January and May; in fact, in May, 60% of subjects in the 600 IU group and 30% in the 4000 IU group had serum 25(OH)D levels below 50 nmol/L.

**Table 2:** Blood test values over time.

Characteristics	Baseline (July 2021) (*n* = 112) Post hoc analysis *p* Values			Follow‐up I (October 2021) (*n* = 103) Post hoc analysis *p* values				Follow‐up II (January 2022) (*n* = 100) Post hoc analysis *p* values					Follow‐up III (May 2022) (*n* = 98) Post hoc analysis *p* values					Two‐way mixed ANOVA
	600 IU *n* = 55	4000 IU *n* = 57	BW groups	600 IU *n* = 50	4000 IU *n* = 53	BW groups	WI group B versus FUI	600 IU *n* = 49	4000 IU *n* = 51	BW groups	WI group B versus FUII	WI group FUI versus FUII	600 IU *n* = 48	4000 IU *n* = 50	BW groups	WI group B versus FUIII	WI group FUII versus FUIII	Within subjects, whole study group, time
Serum 25(OH)D level, nmol/L (recommended level > 75 nmol/L) Mean (SD) Missing values	77.3 (30.09) 0	74.0 (20.08) 0	n.s.	60.6 (14.91) 5	84.6 (23.16) 4	<0.001	600 IU <0.001 4000 IU = 0.01	49.1 (14.00) 6	75.3 (26.96) 6	<0.001	600 IU <0.001 4000 IU n.s.	600 IU = 0.003 4000 IU = 0.03	46.1 (12.88) 7	69.8 (30.82) 7	<0.001	600 IU <0.001 4000 IU n.s.	600 IU n.s. 4000 IU n.s.	**Effects**: Time: **<0.001**
Parathyroid hormone, pmol/L (normal 1.48–7.83 pmol/L) Mean (SD) Missing values	3.29 (1.97) 0	2.67 (1.21) 0	n.s.	3.81 (3.17) 5	2.95 (0.98) 4	n.s.	600 IU n.s. 4000 IU n.s.	4.57 (4.39) 6	3.88 (1.66) 6	n.s.	600 IU <0.001 4000 IU <0.001	600 IU = 0.04 4000 IU = 0.003	4.15 (2.98) 7	3.05 (1.53) 7	n.s.	600 IU = 0.04 4000 IU n.s.	600 IU n.s. 4000 IU = 0.02	**Effects**: Time: **<0.001**
i‐Ca, ionized calcium (normal 1.12–1.32 mmol/L) Mean (SD) Missing values	1.17 (0.03) 0	1.17 (0.04) 0	n.s.	1.23 (0.03) 5	1.24 (0.04) 4	n.s.	600 IU <0.001 4000 IU <0.001	1.20 (0.03) 7	1.21 (0.04) 6	n.s.	600 IU <0.001 4000 IU <0.001	600 IU <0.001 4000 IU = 0.002	1.24 (0.03) 7	1.24 (0.08) 7	n.s.	600 IU <0.001 4000 IU <0.001	600 IU <0.001 4000 IU = 0.006	**Effects**: Time: **<0.001**
Calcium, mmol/L (normal 2.15–2.6 mmol/L) Mean (SD) Missing values	2.53 (0.07) 0	2.54 (0.07) 0	n.s.	2.39 (0.08) 5	2.40 (0.07) 4	n.s.	600 IU <0.001 4000 IU <0.001	2.37 (0.08) 6	2.41 (0.07) 6	n.s.	600 IU <0.001 4000 IU <0.001	600 IU n.s. 4000 IU n.s.	2.34 (0.07) 7	2.37 (0.09) 7	n.s.	600 IU <0.001 4000 IU <0.001	600 IU n.s. 4000 IU = 0.001	**Effects**: Time: **<0.001**

Abbreviations: B, baseline; BW groups, between groups; FUI, Follow‐up I; FUII, Follow‐up II; FUIII, Follow‐up III; IU, international unit; n.s., not significant; SD, standard deviation; WI group, within group.

No significant differences in the PTH, Ca and i‐Ca blood serum levels were found between the study groups at any time point. Significant differences were found in PTH, i‐Ca and Ca blood serum values over time within the study groups. A rise in mean PTH and i‐Ca was found in both study groups, and a decrease in Ca was found in both study groups, with significant differences over time—however, within normative values. The results of blood tests are presented in Table 2.

No significant differences between the study groups at any time points were revealed in the physical fitness tests or hand grip strength tests. However, it appeared that within the study groups, the APFT results had improved at follow–up I compared to baseline. At follow‐up II, compared to baseline, the APFT total scores and sit‐ups in the 4000 IU group and push‐ups in the 600 IU group were significantly improved. A significant decrease was found in the running speed between follow‐up I and follow‐up II in both study groups. Correspondingly, the hand grip strength decreased significantly over time in both study groups. All physical test results are presented in Tables 3 and 4.

**Table 3:** Physical test results during the study period.

Characteristics	Baseline (July 2021) (*n* = 106) Post hoc analysis *p* Values			Follow‐up I (October 2021) (*n* = 106) Post hoc analysis *p* Values				Follow‐up II (May 2022) (*n* = 82) Post hoc analysis *p* Values					Two‐way mixed ANOVA
	600 IU *n* = 50	4000 IU *n* = 56	BW groups	600 IU *n* = 51	4000 IU *n* = 55	BW groups	WI group B versus FUI	600 IU *n* = 41	4000 IU *n* = 41	BW groups	WI group B versus FUII	WI group FUI versus FUII	Within subjects, whole study group, time
APFT scores (0–300) Mean (SD) Missing values	173.3 (52.5) 5	174.1 (60.6) 1	n.s.	219.9 (47.7) 4	220.8 (52.9) 2	n.s.	600 IU <0.001 4000 IU <0.001	195.3 (47.2) 14	204.2 (49.0) 16	n.s.	600 IU n.s. 4000 IU <0.001	600 IU <0.001 4000 IU <0.001	**Effects**: Time: **<0.001**
Running time (min) Mean (SD) Missing values	17:36 (3:29) 8	17:19 (3:39) 3	n.s.	15:09 (1:43) 5	15:01 (1:50) 3	n.s.	600 IU <0.001 4000 IU <0.001	17:22 (3:43) 15	16:52 (3:55) 16	n.s.	600 IU n.s. 4000 IU n.s.	600 IU <0.001 4000 IU <0.001	**Effects**: Time: **<0.001**
Push‐ups (number) Mean (SD) Missing values	45 (15.0) 5	43 (16.3) 1	n.s.	55 (13.0) 4	58 (15.2) 3	n.s.	600 IU <0.001 4000 IU <0.001	54 (12.3) 14	54 (13.7) 16	n.s.	600 IU = 0.001 4000 IU <0.001	600 IU n.s. 4000 IU n.s.	**Effects**: Time: **<0.001**
Sit‐ups (number) Mean (SD) Missing values	48 (13.1) 5	48 (15.2) 1	n.s.	56 (12.5) 4	57 (13.5) 3	n.s.	600 IU <0.001 4000 IU <0.001	54 (12.1) 14	56 (14.4) 16	n.s.	600 IU n.s. 4000 IU = 0.02	600 IU n.s. 4000 IU n.s.	**Effects**: Time: **<0.001**
Passing test yes/no (%) Missing values	11/39 (22) 5	16/40 (29) 1		42/9 (82) 4	43/12 (78) 2			15/26 (37) 14	20/21 (49) 16				

Abbreviations: B, baseline; BW groups, between groups; IU, international unit; FUI, Follow‐up I; FUII, Follow‐up II; FUIII, Follow‐up III; n.s., not significant; SD, standard deviation; WI group, within group.

**Table 4:** Hand grip strength test results during the study period.

Characteristics	Baseline (July 2021) (*n* = 112) Post hoc analysis *p* Values			Follow‐up I (October 2021) (*n* = 102) Post hoc analysis *p* Values				Follow‐up II (January 2022) (*n* = 100) Post hoc analysis *p* Values					Two‐way mixed ANOVA
	600 IU *n* = 55	4000 IU *n* = 57	BW groups	600 IU *n* = 50	4000 IU *n* = 52	BW groups	WI group B versus FUI	600 IU *n* = 49	4000 IU *n* = 51	BW groups	WI group B versus FUII	WI group FUI versus FUII	Within subjects, whole study group, time
Grip test dominant hand (kg), Mean (SD) unit Missing values	46.3 (8.1) 0	44.3 (7.6) 0	n.s.	45.4 (8.0) 5	43.6 (6.8) 5	n.s.	600 IU n.s. 4000 IU n.s.	44.1 (8.2) 6	43.6 (8.4) 6	n.s.	600 IU = 0.04 4000 IU n.s.	600 IU n.s. 4000 IU n.s.	**Effects**: Time: **0.012**
Grip test non‐dominant hand (kg), Mean (SD) unit Missing values	44.3 (8.5) 0	41.4 (7.7) 0	n.s.	42.9 (7.8) 5	43.1 (8.5) 4	n.s.	600IU n.s. 4000 IU = 0.03	41.8 (8.6) 7	40.3 (8.1) 6	n.s.	600 IU = 0.01 4000 IU n.s.	600 IU n.s. 4000 IU <0.001	**Effects**: Time: **<0.001**

Abbreviations: B, baseline; BW groups, between groups; FUI, Follow‐up I; FUII, Follow‐up II; FUIII, Follow‐up III; IU, international unit; n.s., not significant; SD, standard deviation; WI group, within group.

The BMI at baseline was >25 kg/m^2^ in 33 cases, 30–35 kg/m^2^ in 10 cases and 35–40 kg/m^2^ in one case. At no time point time in either group was any significant correlation found between body weight and serum concentration of 25(OH)D except at the baseline in whole study group (Table 5).

**Table 5:** Body weight and vitamin D correlation.

Group	Characteristics	Baseline (July 2021) (*n* = 112)	Follow‐up I (October 2021) (*n* = 103)	Follow‐up II (January 2022) (*n* = 100)	Follow‐up III (May 2022) (*n* = 98)
Total	Body weight Mean (SD) Missing values	79.9 (14.0) 0	80.5 (11.7) 9	80.8 (12.3) 12	82.2 (12.7) 14
	Serum 25(OH)D level, nmol/L Mean (SD) Missing values	75.6 (25.43) 0	72.9 (22.92) 9	62.5 (25.23) 12	58.2 (26.50) 14
	Pearson correlation coefficient between body weight and serum 25(OH)D level (*p* value)	−0.190 (0.045)	−0.026 (0.793)	−0.098 (0.332)	−0.132 (0.196)
600 IU	Body weight Mean (SD) Missing values	81.6 (14.4) 0	82.2 (11.6) 5	82.7 (12.8) 6	83.8 (13.4) 7
	Serum 25(OH)D level, nmol/L Mean (SD) Missing values	77.3 (30.09) 0	60.6 (14.91) 5	49.1 (14.00) 6	46.1 (12.88) 7
	Pearson correlation coefficient between body weight and serum 25(OH)D level (*p* value)	−0.173 (0.207)	−0.098 (0.497)	−0.144 (0.323)	−0.161 (0.275)
4000 IU	Body weight Mean (SD) Missing values	78.3 (13.6) 0	78.8 (11.6) 4	79.1 (11.6) 6	80.7 (11.8) 7
	Serum 25(OH)D level, nmol/L Mean (SD) Missing values	74.0 (20.08) 0	84.6 (23.16) 4	75.3 (26.96) 6	69.8 (30.82) 7
	Pearson correlation coefficient between body weight and serum 25(OH)D level (*p* value)	−0.248 (0.063)	0.157 (0.261)	0.040 (0.781)	−0.065 (0.655)

Abbreviations: IU, international unit; SD, standard deviation.

No side effects of supplementation with vitamin D were registered in either group during the study period.

## DISCUSSION

The main finding of the present study was that vitamin D3 supplementation with a daily dosage of 4000 IU decreased the incidence of vitamin D deficiency compared to taking 600 IU daily in Estonian Army conscripts during a 10‐month period. However, no difference in the physical performance was seen between the study groups.

Vitamin D deficiency is very common in the military community, as shown in several publications [10, 16, 30, 34, 35, 40, 41]. Thus, maintaining sufficient 25(OH)D levels through adequate sun exposure, daily dietary and supplement intake is important, both among the general population and, especially, in the military community. In the military, challenges such as special clothing, lack of natural sun exposure in submarines and shelters, skin camouflage, and high‐level physical stress during training and combat can all potentially affect 25(OH)D levels [16].

Discussions of the normative values of 25(OH)D and suitable supplementation dosages in the general population, and especially in high‐demand populations, are still ongoing. There is a suggestion that the normative value for serum 25(OH)D level for a young, active population should be defined as >75 nmol/L [18]. An even higher upper limit—100 nmol/L—has been suggested by Bischoff‐Ferrari et al. [3]. Another problem is how to reach these suggested values. In the meta‐regression analysis of Cashman et al. [7], a daily vitamin D intake of 930 IU was predicted to maintain a level of 25(OH)D > 50 nmol/L. The Endocrine Society suggests a daily vitamin D supplementation in adults aged 19–50 years to be at least 600 IU to maximize bone health and muscle function. To reach serum levels >75 nmol/L requires a daily supplementation of at least 1500–2000 IU and the upper daily limit of 4000IU should be used in a population with risk of vitamin D deficiency [18]. The Nordic Nutrition Recommendations panel concluded that 97.5% of the population up to 75 years of age need a total intake (food and supplements) of 15 µg (600 IU) to maintain the target level of 50 nmol/L [23].

In an RCT study by Rips et al. [36] of conscripts in the Nordic region, vitamin D supplementation of 1200 IU resulted in only 15% of participants with serum levels of 25(OH)D > 75 nmol/L. This means that achieving the suggested levels is not always possible with moderate (1200 IU) supplementation. In the present study, supplementation with 600 IU seems to be too low to keep serum levels of 25(OH)D sufficient during winter‐ and springtime. Interestingly, in the 4000 IU group in our study, the minimum suggested levels of 50 nmol/L and above were achieved only in 69% of subjects, and sufficient levels of 75 nmol/L and above were achieved in only 37% of subjects in the late springtime. This might be related to the surprisingly low mean 25(OH)D values at baseline at midsummer. Other reasons affecting serum 25(OH)D levels negatively could be high physical exertion [39] and concealing clothing, even with higher doses of vitamin D3 supplementation.

Also, a BMI >30 kg/m^2^ is a risk factor for low 25(OH)D levels [4, 11]. However, no negative correlation between body weight and 25(OH)D blood serum levels could be found in the present study, except for a weak negative correlation for the combined study group at baseline. This might be related to the low number of overweight participants. Based on World Health Organization (WHO) healthy lifestyle recommendations, the normal BMI range is 18.5–24.9 kg/m^2^, pre‐obesity 25–29.9 kg/m^2^, obesity class I 30.03–4.9 kg/m^2^, obesity class II 35.0–39.9 kg/m^2^ and obesity class III >40.0 kg/m^2^, with no account for muscle and fat distribution in body composition [1]. However, in some cases, BMI might not be the best tool to assess physical performance capability. Potter et al. [31] found that a BMI of 33.0 in fit marines was not related to the amount of fat, but rather to muscle, and these subjects were considered physically fit for extreme conditions. In the present study, a rise in body mass was found over time in both study groups, and most of the participants were considered to be people with pre‐obesity based on the WHO classification. Unfortunately, no body composition assessments were performed during the study.

There is still controversy surrounding whether vitamin D supplementation affects physical performance in demanding populations such as professional athletes or military personnel. The Endocrine Society consensus panel has considered that there is no target value for serum 25(OH)D concentration with regard to muscle strength or function and physical performance [9]. Interestingly, at the same time, there is proof of the importance of vitamin D to muscle cells [25]. In line with the Endocrine Society consensus, a short‐term placebo‐controlled study by Menon et al. [29] found no effect on cardiorespiratory fitness, or muscle strength, during 19 weeks of basic military training in a group of 90 males. In an RCT by Savolainen et al. [37], no positive effect of high‐dosage (8000 IU) vitamin D supplementation for 12 weeks was found on muscle strength in vitamin D‐deficient young males.

Another large study of 967 young, healthy military recruits, both males and females, where no supplementation was given, found no correlation in terms of muscle strength or power, but a positive correlation between endurance performance and 25(OH)D levels. From the same study group, 137 selected men entered a 12‐week placebo‐controlled trial; in this trial, the intervention group received supplementation of 1000 IU for four weeks, followed by 400 IU for 8 weeks, while the control group received placebo for 12 weeks. No differences between the study groups were found in terms of physical or endurance performance [6]. The influence of vitamin D on physical performance was also studied by Rips et al. [35], in their study of 98 conscripts over a 10‐month military service period, they could not find any negative effects, even with critically low 25(OH)D levels—under 25 nmol/L. On the other hand, Heileson et al. found that vitamin D was positively associated with all important outcomes, such as strength, and muscular and cardiorespiratory endurance [15]. Barringer et al. [2], in a study of 100 soldiers, found vitamin D to be positively associated with push‐up performance during the APFT.

In the present study, two different physical performance tests were used. In the APFT test, it was found that conscripts were able to increase their muscle strength in the sit‐up and push‐up tests and their cardiovascular capacity in the form of the running speed test at follow‐up I in both groups. However, no differences between the study groups were found. Interestingly, in the last test session in May (follow‐up II), a significant decrease in the results, especially in running speed and total scores, compared to follow–up I was seen in both study groups. The reason for this is unclear, but weather conditions or lack of motivation just before discharge from service could influence the results. The same was found for the hand grip strength test, with a significant decrease in the results in the 600 IU group at follow‐up II compared to baseline. It therefore appears that the increase and decrease in test scores is based on the training effect, rather than vitamin D supplementation.

The results of the present study are different compared to those of a large population‐based study involving 73,699 participants in which a higher serum concentration of 25(OH)D was found to be associated with a greater grip test strength in both hands [27]. This might be related to the fact that maximum hand grip strength development occurs at a later age than in the present study [26]. Based on the present study, 10 months of vitamin D supplementation does not influence hand grip strength in young males.

In terms of PTH, i‐Ca, and Ca in the serum, minimal changes outside of normative values were found with no signs of side effects. Low 25(OH)D status increases PTH levels and calcium release from the bone [33]. In the present study, an increase in PTH was found in the 600 IU group, which had lower mean values of 25(OH)D, and more stable PTH values were found in the 4000 IU group, which had higher vitamin D values. Interestingly, seasonal variation in PTH was described in a large cohort study by Shen et al. [38]. Fluctuations of PTH levels are also related to diet, time of day, renal function, physical activity and most importantly the suppression of PTH levels by optimal serum levels of 25(OH)D [3]. The findings of higher levels of PTH in the 600 IU group with lower levels of 25(OH)D are in line with previous studies [3, 33].

According to the present study, it seems that in a population with high physical demands, a higher (4000 IU) vitamin D supplementation dosage is needed, and a low dosage (600 IU) supplementation has little effect on keeping the serum 25(OH)D levels high enough during winter‐ and springtime. Contrary to some other studies [2, 15, 27], no effect of vitamin D supplementation between the 600 IU and 4000 IU groups was found in terms of physical performance in male conscripts.

Still, long‐term vitamin D deficiency could have negative effects on general health overall, and, therefore, the authors suggest that in a physically active population, measurement of serum 25(OH)D level could be performed during winter‐ and springtime to find out if supplementation is needed to prevent seasonal vitamin D deficiency. Based on findings in the present study, a 600 IU supplementation during winter‐ and springtime in the Nordic regions is insufficient. Supplementation during 10 months with 4000 IU appears to have no negative side effects of young men with increased physical demands and can therefore be recommended. The future perspective is to perform a large multicenter study from different geographic areas to further understand vitamin D supplementation needs in a physically high‐demand group such as conscripts.

The strengths of the present study are its randomized and blinded design and that the long 10‐month follow‐up period included all seasons. All physical activities, clothing, seasonal influences, food consumption, age, and sex of participants were also standardized. The limitations of the study include the relatively small groups compared to larger cohort studies, the relatively high dropout rate, the absence of a placebo group, a relatively high number of missed physical tests, the high number of dropouts, different physical test times and a possible non‐compliance of daily vitamin D supplementation, in spite of vigorous monitoring. Furthermore, lack of a body composition assessment, and no hand grip test was performed in late spring.

## CONCLUSION

A 10‐month vitamin D supplementation with 4000 IU decreased the incidence of vitamin D deficiency (<75 nmol/L) in young, male army conscripts during wintertime, but no differences in physical performance were found compared to 600 IU supplementation.

## AUTHOR CONTRIBUTIONS

Leho Rips: Designed and conducted research; analyzed data; writing of paper. Alar Toom: Analyzed data; writing of paper. Rein Kuik: Designed research. Ahti Varblane: Designed research; conducted research. Hanno Mölder: Conducted research. Ragnar Kibur: Conducted research. Marika Laidvere: Conducted research. Mart Kull: Analyzed data; writing of paper. Jüri‐Toomas Kartus: Design of study; primary responsibility for final content; writing of paper; statistical analyses. Helena Gapeyeva: Designed research; conducted research. Madis Rahu: Primary responsibility for final content; writing of paper.

## CONFLICT OF INTEREST STATEMENT

Leho Rips: Consultant Orthopedic Surgeon of Estonian Defence Forces. Alar Toom, Rein Kuik, Marika Laidvere, Mart Kull, Helena Gapeyeva, and Madis Rahu: No conflict of interest. Ahti Varblane, Hanno Mölder, and Ragnar Kibur: Doctor of Estonian Defence Forces. Jüri‐Toomas Kartus: Lecturing for ConMed, Sweden.

## ETHICS STATEMENT

The study was approved by the Research Ethics Committee of the University of Tartu nos. 323/T‐3, 337M‐25, and 341M‐12. Trial registration: ClinicalTrials.gov Identifier: NCT04939636. Prospectively registered 13 October 2020. Only those who volunteered to participate in the study and provided signed informed consent were included.

## Data Availability

The data sets generated and/or analyzed during the current study are available in the data, University of Tartu Library repository, https://doi.org/10.23673/re-368.
